# The Department of Defense Global Respiratory Pathogen Surveillance Program: Its Impact on Public Health, from the U.S. Armed Forces to Global Health

**Published:** 2025-04-20

**Authors:** William E. Gruner, Laurie S. DeMarcus, Jeffrey W. Thervil, Bismark Kwaah, Whitney N. Jenkins, Amy L. Bogue, Tamara R. Hartless, Anthony S. Robbins, James F. Hanson, Jimmaline J. Hardy, Deanna M. Muehleman, Anthony C. Fries, Elizabeth A. Macias

**Affiliations:** JYG Innovations, LLC, Dayton: Mr. Gruner, Dr. Muehleman, Dr. Hardy; Innovative Element, LLC, Beavercreek, OH: Ms. DeMarcus, Mr. Thervil, Mr. Kwaah, Ms. Jenkins, Ms. Bogue, Ms. Hartless; U.S. Defense Health Agency Armed Forces Health Surveillance Division Air Force Satellite: Lt Col Robbins; Defense Centers for Public Health–Dayton, Wright-Patterson AFB, OH: Dr. Fries, Dr. Macias, Mr. Hanson, Maj Watts

## Abstract

The U.S. Department of Defense Global Respiratory Pathogen Surveillance Program (DoDGRPSP) has supported the prevention of respiratory illness in the U.S. Armed Forces since 1976, supported by the Armed Forces Health Surveillance Division Global Emerging Infections Surveillance (AFHSD-GEIS) branch of the Defense Health Agency (DHA) since 1997. DoDGRPSP utilizes a global network of sentinel sites and partner laboratories to collect respiratory surveillance data and share its findings with the U.S. Department of Defense (DOD) and installation stakeholders. Several significant findings have resulted from the program in the last decade, including novel influenza detections, outbreak characterizations, and early detection of SARS-CoV-2 variants. The program collaborates with other DOD and government entities to inform public health decisions, including vaccine effectiveness estimates, phylogenetic analyses, and antigenic characterizations to the U.S. Food and Drug Administration to aid selection of influenza strains for subsequent U.S. vaccines. DoDGRPSP adapts to changes in emerging pathogens, evolution of known pathogens, advancements in respiratory pathogen testing assays and instruments, new analytical methods, and new sequencing technologies. The program continues to provide continuous respiratory pathogen surveillance data, vaccine effectiveness estimates, and sequence data analyses in reports and peer-reviewed publications to DOD, government, and global partners.

The U.S. military plays a crucial role in combatting global respiratory illnesses. The close quarter, high stress environments of training stations that house recruits from a wide range of geographic areas constitute ideal situations for the introduction, spread, and mutation of respiratory pathogens. Conditions are similar at deployed locations, but with added risk of service member exposure to novel pathogens not encountered in the U.S. The regular movement of military personnel through deployments and routine changes of station facilitates wide diffusion of pathogens across an enormous geographic range and makes isolation of emergent pathogens extremely difficult.

The global network of U.S. military installations, in addition to providing locations of deployment and coordination with foreign military units, also afford extraordinary capacity for identifying and characterizing respiratory illnesses. The U.S. Department of Defense Global Respiratory Pathogen Surveillance Program (DoDGRPSP), currently based at Wright-Patterson Air Force Base (AFB) in Dayton, Ohio, is a cornerstone of U.S. Department of Defense (DOD) respiratory disease surveillance. DoDGRPSP currently relies upon a surveillance network of 115 active sentinel sites in addition to other participating sites, deployed locations, and partner laboratories.

DoDGRPSP was established in 1976 as part of the U.S. Air Force School of Aerospace Medicine (USAFSAM) at Brooks AFB in San Antonio, Texas. Then known as “Project Gargle,” the program initially collected specimens from Lackland AFB, which conducted Air Force basic training. Over time, the program expanded its specimen collection from military and Coast Guard sites within the contiguous U.S. (CONUS) as well as outside the contiguous U.S. (OCONUS). Twenty years after the program was founded, the 1996 Presidential Decision Directive, National Science and Technology Council-7, tasked the DOD with enhancing its mission by increasing global surveillance for emerging infectious disease, improving research and training, engaging with international partners, and strengthening public outreach to address emerging infectious diseases. In response to this directive, the following year the DOD Global Emerging Infections Surveillance (DOD-GEIS) program was established.

In the years after DOD-GEIS was established, OCONUS DOD laboratories expanded their reach, capability, and coordination with CONUS surveillance systems, which at the time primarily comprised USAFSAM and the Naval Health Research Center (NHRC) in San Diego, California. By 2006, the 2 programs had expanded to include all DOD services, with increased surveillance networks and standardized force health protection communications to CONUS and OCONUS facilities. In 2011, DOD-GEIS was transferred to the (now) Armed Forces Health Surveillance Division (AFHSD), and USAFSAM was relocated to Wright-Patterson AFB. ^1^ More recent DHA reorganization shifted DoDGRPSP authority to the Defense Centers for Public Health–Dayton (DCPH-D).

Year-round data collection from respiratory testing at USAFSAM / DCPH-D allows DoDGRPSP to create seasonal epidemiological curves for influenza, SARS-CoV-2, and numerous other respiratory pathogens. These curves encompass cumulative, regional, or installation-specific data, allowing leadership, health care providers, or public health employees within participating sites, Combatant Commands (COCOMs), or the DOD to determine risk levels, causative agents of respiratory illness, or mount appropriate public health measures.

## Collaborations

DoDGRPSP collaborates with other DOD as well as non-DOD government agencies to ensure that surveillance data collected are efficacious for force health protection. The program routinely communicates with government partners to maintain up-to-date testing and sequencing assays, assess currently circulating strains and analyses, evaluate naming conventions, and share data or specimens that may be unique or propitious. These consultations occur with regularity throughout the year but intensify in the months preceding the annual World Health Organization (WHO) and Vaccines and Related Biological Products Advisory Committee (VRBPAC) meetings for the Northern Hemisphere and U.S. influenza strain recommendations, respectively.

One of the signal functions of DoDGRPSP is the preparation and presentation of the surveillance data for U.S. influenza vaccine strain recommendations. Each year, the U.S. Food and Drug Administration (FDA) VRBPAC ^2^ meets to discuss the annual influenza vaccine. In addition to the Centers for Disease Control and Prevention (CDC), a DOD representative presents mid-season surveillance results, vaccine effectiveness (VE) estimates, phylogenetic data, and antigenic cartography information to the committee, which then votes to accept or reject strain recommendations made by WHO based on season-to-date VE of current strains, sub-type dynamics, and changes to circulating influenza virus clades. ^3^

For antigenic characterization data, DoDGRPSP partners with the Navy Medical Research Command (NMRC) in Silver Spring, Maryland, to share specimens and data that are relevant to diverse strains of circulating influenza and SARS-CoV-2. Antibodies raised against current and candidate vaccine strains are tested against circulating strains to comparatively test levels of inhibition and determine which strains could provide broadest protection. These data are visually modeled using anti-genic cartography and then presented to VRBPAC.

DoDGRPSP also collaborates with agencies such as the National Center for Biotechnology Information (NCBI), the Walter Reed Army Institute of Research (WRAIR), and the Infectious Disease Clinical Research Program (IDCRP) on, for example, database nomenclature consultation, ^4^ influenza vaccine breakthrough sieve analysis studies (ongoing, unpublished), the ARIA (Acute Respiratory Illness at Academies) study (ongoing, unpublished), and the PAIVED (Pragmatic Assessment of Influenza Vaccine Effectiveness in the DOD) study. ^5^

## Surveillance Network

A crucial part of any successful surveillance program is a robust network of collection sites and personnel not only capable but willing to participate. The DoDGRPSP team relies on a program network of sentinel, participating, deployed, and partner sites in multiple ways. Any breaks in a surveillance network risk missed important surveillance data, skewed data analyses, and a distorted picture, in scale or scope, of an emerging outbreak or seasonal trends.

At the beginning of each season, an approved program memorandum outlines site participation criteria and lists the sentinel sites selected to submit samples for the season. The list of sites is for broad, evenly distributed geographic coverage, while taking into consideration installation populations, capabilities, tri-service coverage, and past program participation. Sentinel sites may be removed from the list if they lack the resources to participate (e.g., freezers, facilities, personnel), or conversely, can be added based on ability and willingness to participate. OCONUS partner laboratories, which generate their own data through sample collection, testing, and sequencing, can help mitigate geographic gaps in surveillance data.

Sites participating in DoDGRPSP are asked to submit 6 to 10 respiratory specimens per week from patients meeting an influenza-like or COVID-19-like illness case definition (i.e., fever at or above 100.4°F and cough or sore throat ^6^ ) or 1 or more symptoms associated with influenza or COVID-19, although clinical suspicion of respiratory illness also qualifies for submission. In response to the 2024 increase in cattle and human cases of avian influenza A(H5N1), conjunctivitis with known exposure to agricultural animals or humans infected with influenza A(H5N1) was added as a symptom category. ^7^ Participating sites are also asked to have personnel submit a questionnaire that collects patient demographic, symptomatic, and vaccination history information. These questionnaires allow the program to perform VE analysis, as well as reporting or conducting studies based on cumulative patient demographic or symptomology associated with laboratory results.

Education and training are vital components of maintaining a surveillance network. At the beginning of each season, online training sessions are conducted for the surveillance sites, at multiple times and dates to accommodate schedules and global time zones. Each training is followed by a question-and-answer session. DoDGRPSP team members also conduct selected site visits each year, which provide direct interactions whereby team members can learn about the processes and workflows of individual installations while providing potential solutions to barriers of participation, often based on experiences from other sites. These in-person meetings lead to closer relationships with points of contact that can help bolster participation. The DoDGRPSP team reaches out to sites with low participation as a reminder of compliance and to help mitigate any problems that may be hindering sample submission such as collection kit supplies or MHS GENESIS ordering issues.

## Specimen Testing

Specimens collected through the DoDGRPSP surveillance network are clinically tested in the College of American Pathologists (CAP)-accredited Epidemiology Laboratory in the Public Health department at USAFSAM / DCPH-D. Prior to 2018, specimens were initially tested using CDC influenza A / B and A subtype RT-PCR assays, ^8^ with influenza-negative specimens tested on the BioFire FilmArray Respiratory Panel (RP), ^9^ which tests for additional pathogens listed in **Table 1** . If an influenza A specimen could not be sub-typed on either assay, then CDC influenza A / H5 and A / H7 subtype assays were performed. A positive A / H5 or A / H7 would be sent to the CDC for confirmation, although to date no positives have been identified. Selected specimens undergo viral culture to grow isolates and characterize pathogens **(Table 1)** . Sanger sequencing was performed on isolates from influenza-positive specimens for the hemagglutinin (HA), neuraminidase (NA), and matrix protein (MP) genes.

**TABLE 1.** Respiratory Panel Testing at USAFSAM / DCPH-D Epidemiology Laboratory, 2014–Present

Pathogen	Type of Testing		
	BioFire FilmArray Respiratory Panel 2014-2018	Luminex NxTag Respiratory Pathogen Panel 2018-Present	Viral Culture
Viral			
adenovirus	✓	✓	✓
human coronavirus 229E	✓	✓	
human coronavirus HKU1	✓	✓	
human coronavirus NL63	✓	✓	
human coronavirus OC43	✓	✓	
influenza A H1	✓	✓	✓
influenza A H1-2009	✓	✓	✓
influenza A H3	✓	✓	✓
influenza B	✓	✓	✓
human metapneumovirus	✓	✓	
parainfluenza 1-3	✓	✓	✓
respiratory syncytial virus (A, B)	✓	✓	✓
rhinovirus/enterovirus	✓	✓	✓
human bocavirus		✓	
Bacterial			
*Bordetella pertussis*	✓		
*Chlamydophila pneumoniae*	✓	✓	
*Mycoplasma pneumoniae*	✓	✓	

Abbreviations: USAFSAM, U.S. Air Force School of Aerospace Medicine; DCPH-D, Defense Centers for Public Health–Dayton.

Beginning in 2018, the Luminex NxTag Respiratory Pathogen Panel (RPP) was adopted, and the testing algorithm was adjusted to perform RPP first, then CDC influenza A / B and A subtype RT-PCR on un-subtyped influenza specimens. The NxTag RPP allows high throughput testing for the pathogens listed in **Table 1** . Next-Generation Sequencing (NGS) was also adopted in 2018, allowing whole genome sequencing of influenza-positive specimens using an original specimen rather than cultured isolates. When the COVID-19 pandemic began, SARS-CoV-2 PCR was adopted, as well as whole genome sequencing of other selected respiratory-positive specimens.

An average of 5,760 (range 4,915–6,338) specimens were tested each season from 2014 until 2018, when the CDC influenza assays were the primary testing procedure, followed by FilmArray RP. During the same period, the average number of influenza-positive specimens sequenced was 1,363 (range 1,080–1,698), using Sanger sequencing.

After changing the primary testing method to the NxTag RPP, the number of specimens tested increased to 12,305 in the 2018-2019 season. The average number of specimens increased in the following seasons, but those numbers were skewed by the sheer number of SARS-CoV-2 tests performed during the 2020-2021 and 2021-2022 seasons. With implementation of NGS in 2019, the number of influenza-positive specimens sequenced increased to 3,059, for the 2018-2019 season.

Due to the COVID-19 pandemic, the number of influenza specimens sequenced each season has varied widely. The average number of SARS-CoV-2 positives sequenced per season from 2020 to 2024 was 5,574 (range 1,361–12,118) **(Table 2)** .

**TABLE 2.** Testing Data Processed at DoDGRPSP per Influenza Season, 2014–2024

	Season									
	2014–2015	2015–2016	2016–2017	2017–2018	2018–2019	2019–2020	2020–2021	2021–2022	2022–2023	2023–2024
Clinical Site										
Tested at USAFSAM / DCPH-D	6,338	4,915	6,027	9,987	12,305	24,788	60,323	30,085	6,831	9,197
Data from LRMC / EUCOM	2,445	1,439	1,617	2,451	2,119	2,345	37,800	37,370	15,903	3,379
Data from Incirlik	—	—	—	—	—	—	32	1,830	929	449
Data from BAMC	—	—	—	—	1,156	1,710	—	—	—	—
Sequencing										
Influenza sequenced	1,080	1,312	1,698	2,363	3,059	3,070	21	1,485	975	1,269
SARS sequenced	—	—	—	—	—	—	7,199	12,118	1,361	1,617

Abbreviations: DoDGRPSP, Department of Defense Global Respiratory Pathogen Surveillance Program; USAFSAM, U.S. Air Force School of Aerospace Medicine; DCPH-D, Defense Centers for Public Health–Dayton; LRMC, Landstuhl Regional Medical Center; EUCOM, European Command; BAMC, Brooke Army Medical Center; SARS, severe acute respiratory syndrome.

In addition to testing at USAFSAM/DCPH-Dayton, DoDGRPSP imputes surveillance data through data pulls and questionnaires from Landstuhl Regional Medical Center (LRMC) in Germany, Incirlik Air Base in Turkey, as well as Brooke Army Medical Center (BAMC) in Texas. Additional influenza sequence data have been supplemented through partnerships with global GEIS network partner laboratories (which are listed in the Acknowledgments).

## In-Depth Pathogen Characterization

When respiratory infections are abnormally high at an installation, within a geographic region, or when a site notifies DCPH-D about an outbreak, DoDGRPSP performs additional testing and characterization. While the collection of laboratory testing data along with demographic, syndromic, and vaccination data from questionnaires are essential, even the most complete data sets do not tell a complete story. Influenza type and subtype data alone do not provide insights into how strains may be mutating, how closely they are related to the current vaccine strain, or what strains would work best in the next vaccine formulation. Sequencing can answer many of these questions.

Each of the 8 influenza gene segments can provide some information about how a particular virus may respond to vaccine-induced antibodies, antiviral therapeutics, or the host immune system. Because they are the 2 surface proteins that interact with cellular receptors and antibodies, hemagglutinin (HA) and neuraminidase (NA) are the primary targets for determining vaccine coverage and are utilized to assign an influenza virus to a genetic grouping, or clade **(** **Figure 1** **)** . Influenza viruses in the same clade are often alike antigenically, therefore differences in circulating clades can provide insights into potential vaccine protection. Specific mutations to antigenic sites, the receptor binding site, or glycosylation motifs may alter vaccine effectiveness, ^10^ antiviral efficacy, ^11^ testing capabilities, ^12^ and the course of disease. ^13^ Additionally, mutations occurring in the remaining 6 gene segments have been known to affect antiviral resistance ^11^ and virulence. ^13^

**FIGURE 1a.**
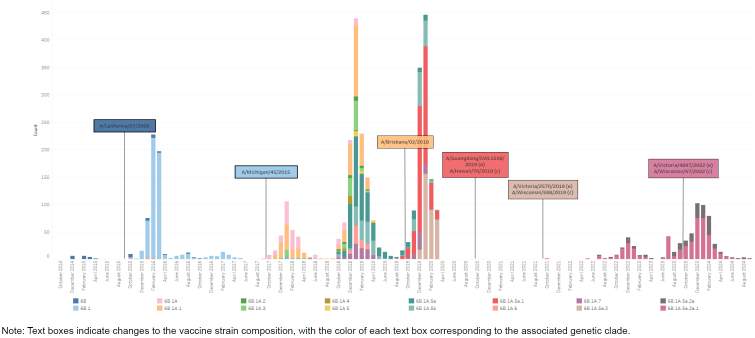
Influenza Clades for Influenza Subtype A(H1N1)pdm09, Influenza Subtype A(H3N2), Influenza Subtype B / Victoria, and Influenza Subtype B / Yamagata, October 2014–August 2024 Influenza Subtype A(H1N1)pdm09

**FIGURE 1b.**
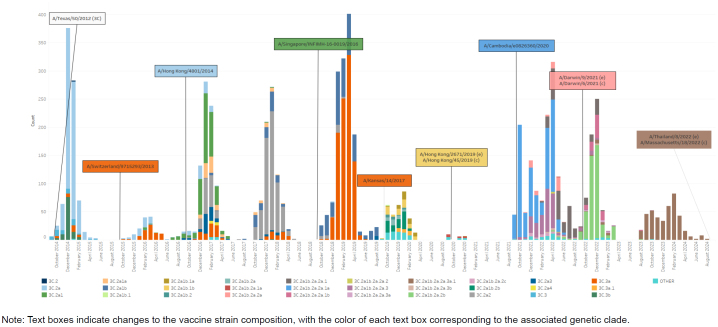
Influenza Clades for Influenza Subtype A(H1N1)pdm09, Influenza Subtype A(H3N2), Influenza Subtype B / Victoria, and Influenza Subtype B / Yamagata, October 2014–August 2024 Influenza Subtype A(H3N2)

**FIGURE 1c.**
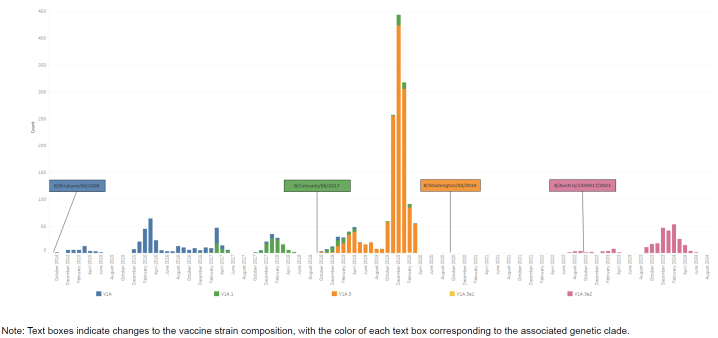
Influenza Clades for Influenza Subtype A(H1N1)pdm09, Influenza Subtype A(H3N2), Influenza Subtype B / Victoria, and Influenza Subtype B / Yamagata, October 2014–August 2024 Influenza Subtype B / Victoria

**FIGURE 1d.**
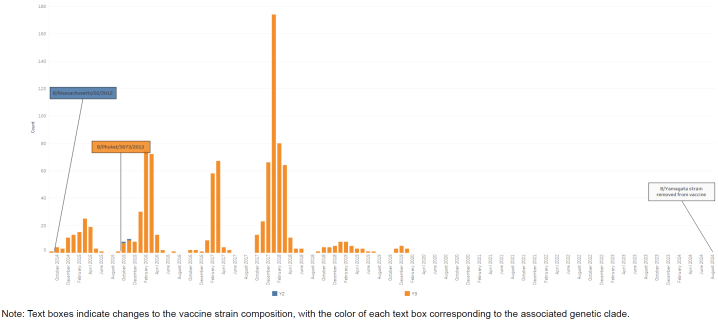
Influenza Clades for Influenza Subtype A(H1N1)pdm09, Influenza Subtype A(H3N2), Influenza Subtype B / Victoria, and Influenza Subtype B / Yamagata, October 2014–August 2024 Influenza Subtype B / Yamagata

Epidemiologists at DCPH-D calculate influenza VE at both mid-season and end of season **(** **Figure 2** **)** by comparing vaccinated and unvaccinated patients in a case-control study, in which laboratory-confirmed influenza-positive specimens serve as cases and laboratory-confirmed influenza-negative specimens serve as controls. Crude and adjusted odds ratios (ORs) are calculated using logistic regression, and VE is calculated as (1 – OR)*100. When case numbers are high enough to yield statistically significant results, comparisons can be made among age groups, influenza subtypes, or in rare instances, genetic clades.

**FIGURE 2a.**
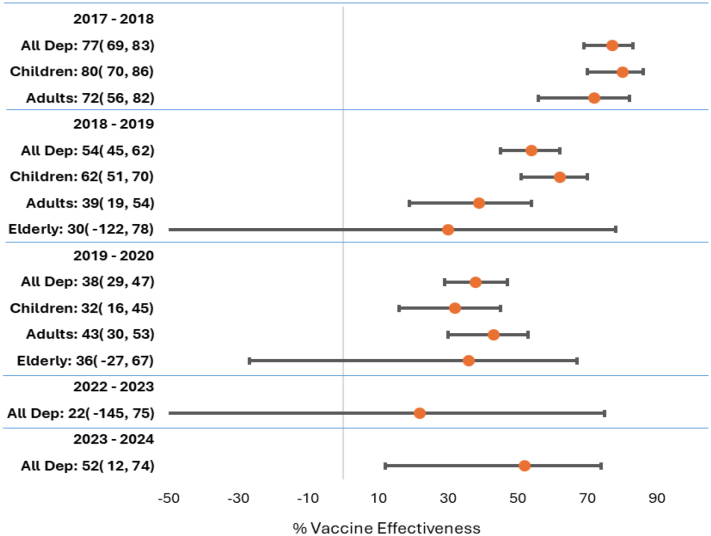
Vaccine Effectiveness, Influenza Subtype A(H1N1)pdm09, Influenza Subtype A(H3N2), and Influenza B, 2017–2024 Influenza Subtype A(H1N1)pdm09

**FIGURE 2b.**
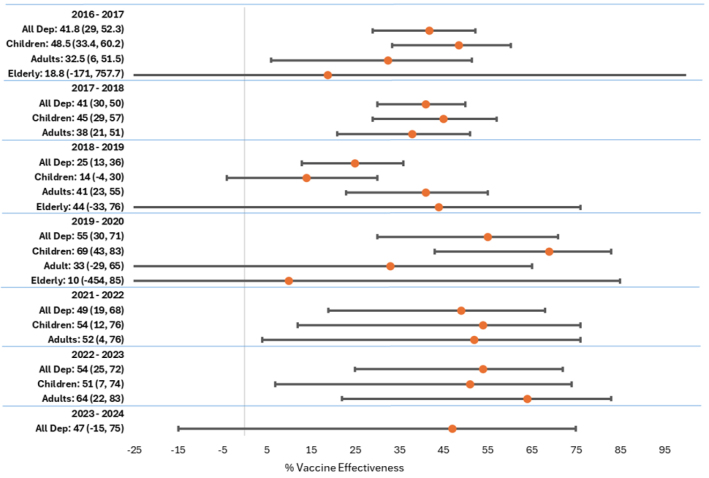
Vaccine Effectiveness, Influenza Subtype A(H1N1)pdm09, Influenza Subtype A(H3N2), and Influenza B, 2017–2024 Influenza Subtype A(H3N2)

**FIGURE 2c.**
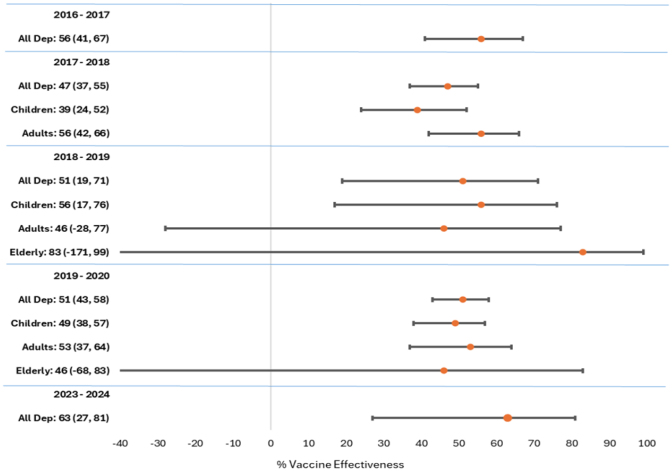
Vaccine Effectiveness, Influenza Subtype A(H1N1)pdm09, Influenza Subtype A(H3N2), and Influenza B, 2017–2024 Influenza B

With the expansion to multiplex PCR testing, the program expanded its sequencing efforts to other respiratory pathogens, including SARS-CoV-2 viruses, to monitor for emerging variants, changes to variant proportions, and mutations posing possible threats to public health, either through host immune evasion, decreased response to vaccine-induced antibodies, or decreased effectiveness of therapeutic measures.

## Impacts on Public Health

DoDGRPSP has detected and reported several significant findings over the past decade, including influenza clades linked to vaccine mis-matches, influenza swine variants, early detection of SARS-CoV-2 lineages, and in-depth characterizations of respiratory pathogen outbreaks.

DoDGRPSP surveillance findings are reported through the program's Common Access Card (CAC)-enabled dashboard, ^14^ which includes routine weekly and cumulative season reports. These reports contain summaries, trends, visualizations and interpretations of results, specimen submissions by site, and symptomatic, immunization, demographic, and sequencing data. Weekly reports are published on the dashboard as well as emailed to DOD, network partners, and entities and individuals who requested entry in the distribution list.

The program dashboard provides aggregated results for all data and regions, while sentinel sites can view their specific surveillance results along with sample and questionnaire submission numbers. Influenza and SARS-CoV-2 sequencing results were recently added to the dashboard, offering more detailed insight on circulating strains. An electronic questionnaire developed through the program dashboard ^14^ now allows more seamless pairing of questionnaire data to surveillance specimens; site participants may prefer this option to paper questionnaires.

Resources such as program information, training videos, contact information, and links to the electronic or printable PDF questionnaire are available on the dashboard. Additional dashboard and report changes will not only make communicating surveillance data more efficient and timelier, but make data more easily digestible with a ‘bottom line up front’ (BLUF) approach and modernized tables and figures.

Data collected by the program are also shared with broader scientific and public health communities, such as with the CDC, through the Public Health Laboratory Interoperability Project (PHLIP). ^15^ Twice a year VE and sequencing data are contributed to the WHO Global Influenza Vaccine Effectiveness (WHO-GIVE) report, prior to the annual VRBPAC meeting for the U.S. influenza vaccine (Northern Hemisphere) strain selection, and at the end of the season for the Southern Hemisphere strain selection.

A cumulative report of the surveillance data is published in *MSMR* ^16 - 19^ and on DTIC. The program publishes specific studies, most recently a SARS-CoV-2 re-infection study in the November 2024 supplement of *Emerging Infectious Diseases* , ^20^ and presents posters at conferences including the Association of Public Health Laboratories, the American Society for Microbiology, the American Society of Virology, the American Society of Tropical Medicine and Hygiene, the International Conference on Emerging Infectious Diseases, and the Military Health System Research Symposium. Sequence data are de-identified and uploaded to the Global Initiative on Sharing All Influenza Data (GISAID) ^21^ repository and NCBI Gen-Bank, ^22^ and Sequence Read Archive (SRA) repositories under USAFSAM / DCPH-D Bioprojects. ^23^

DoDGRPSP recognizes the wealth of its data and potential for retrospective analyses, to inform studies and publications that can further contribute to the scientific community and advance pathogen mitigation efforts. Laboratory results, sequencing data, and syndromic and vaccination records from questionnaires in DoDGRPSP databases hold untold potential for valuable future analyses and conclusions.

DoDGRPSP is continuously evaluating improved testing platforms, procedures, analyses, and use of its surveillance data. The program assesses new instruments and assays to optimize throughput, budget, and data relevance, in addition to enhanced data reporting for optimal impacts. With the ever-changing universe of respiratory pathogens, DoDGRPSP seeks improved capacities for adaptation and response to emerging pathogens as quickly as possible, for timely and meaningful data and analysis reporting.
